# Adverse events profile of oral corticosteroids among asthma patients in the UK: cohort study with a nested case-control analysis

**DOI:** 10.1186/s12931-018-0742-y

**Published:** 2018-04-27

**Authors:** Marlene Bloechliger, Daphne Reinau, Julia Spoendlin, Shih-Chen Chang, Klaus Kuhlbusch, Liam G. Heaney, Susan S. Jick, Christoph R. Meier

**Affiliations:** 1 Basel Pharmacoepidemiology Unit, Division of Clinical Pharmacy and Epidemiology, Department of Pharmaceutical Sciences, University of Basel, Basel, Switzerland; 2 Hospital Pharmacy, University Hospital Basel, Basel, Switzerland; 3 Genentech, Inc., South San Francisco, CA USA; 4 F. Hoffmann-La Roche, Basel, Switzerland; 5 Wellcome-Wolfson Institute for Experimental Medicine, Queens University Belfast, Belfast, Northern Ireland; 6 Boston Collaborative Drug Surveillance Program, Boston University School of Public Health, Lexington, MA USA; 7 Basel Pharmacoepidemiology Unit, Hospital Pharmacy, University Hospital Basel, Spitalstrasse 26, CH-4031 Basel, Switzerland

**Keywords:** Clinical practice research datalink, Asthma, Corticosteroids, Adverse events, Observational study

## Abstract

**Background:**

To evaluate the adverse events profile of oral prednisolone among adult asthma patients in the UK.

**Methods:**

Using data from the UK-based Clinical Practice Research Datalink, we conducted a series of cohort studies to quantify incidence rates and incidence rate ratios, and a series of nested case-control analyses to estimate crude and adjusted odds ratios, of 11 different potential corticosteroid-related adverse events (bone-related conditions, hypertension, peptic ulcer, severe infections, herpes zoster, diabetes mellitus type 2, cataract, glaucoma, chronic kidney disease, affective disorders, and cardiovascular events).

**Results:**

Between 165,900 and 269,368 asthma patients were included in each of the 11 cohorts, of whom between 836 and 16,192 developed an outcome of interest. Incidence rates per 1000 person-years of potential corticosteroid-related adverse events in patients with new current use of oral prednisolone ranged from 1.4 (95% confidence interval [CI], 1.0–1.8) for peptic ulcer to 78.0 (95% CI, 74.8–81.2) for severe infections. After adjusting for confounding, current oral prednisolone use was most strongly associated with an increased risk of severe infection, compared with non-use of prednisolone; OR 2.16 (95% CI, 2.05–2.27). There were smaller elevated risks of peptic ulcer, affective disorders, and cataract at higher doses, and marginally increased risks of herpes zoster, cardiovascular events, diabetes mellitus type 2, and bone related conditions, compared with non-use of prednisolone. We did not observe an association between oral prednisolone use and glaucoma, chronic kidney disease, or hypertension.

**Conclusion:**

Oral prednisolone use is associated with infections, gastrointestinal, neuropsychiatric, ocular, cardiovascular, metabolic, and bone-related complications among adult asthma patients.

**Electronic supplementary material:**

The online version of this article (10.1186/s12931-018-0742-y) contains supplementary material, which is available to authorized users.

## Background

Asthma is among the most common chronic diseases worldwide and poses a substantial public health burden [1, 2]. In the United Kingdom (UK), one in 12 adults requires asthma treatment, and the disease accounts for more than 1000 deaths a year [3].

The Global Initiative for Asthma (GINA) and the British Thoracic Society/Scottish Intercollegiate Guidelines Network (BTS/SIGN) guidelines for asthma management include a stepwise treatment approach based on use of inhaled corticosteroids (ICS) with or without inhaled long-acting beta-adrenoceptor agonists (LABA) [4, 5]*.* For patients with severe asthma not controlled by ICS and LABA, guidelines include low-dose oral corticosteroids (OCS) as add-on treatment to mitigate inflammation, relieve symptoms, and prevent exacerbations.

OCS use has been linked to a variety of adverse events including osteoporosis and subsequent bone fractures, peptic ulcer, susceptibility to infections, hyperglycaemia, hypertension, ocular complications, cardiovascular events, and neuropsychiatric disorders [6–9]. Two recently conducted cross-sectional studies among asthma patients based on United States (US) commercial claims data and data from UK primary and secondary care databases, respectively, suggested relevant associations between frequent OCS use and various potential corticosteroid-related adverse events [10, 11]. However, the temporal relationship between these disorders and OCS exposure could not be established. Three retrospective cohort studies based on different US claims databases also documented a potentially increased risk for corticosteroid-related diseases in asthma patients who used systemic corticosteroids compared with non-users; additionally, the risk increased with increasing daily OCS dose [12–14]. Most of these studies restricted OCS exposed groups to severe asthma patients with frequent OCS use [10, 11], or with an initial period of continuous systemic corticosteroid use at high daily doses (≥5 mg prednisolone or equivalent) [12, 13]. The most recently published cohort study included asthma patients with different disease stages and assessed the association between the frequency of OCS prescriptions issued per year and various adverse outcomes [14]. None of these studies investigated absolute risk estimates of potentially corticosteroid-related adverse events among asthma patients with or without OCS use.

In the present study, we examined the risk of OCS-related adverse events in a large population of UK patients enrolled in the well-validated Clinical Practice Research Datalink (CPRD). We included patients with different stages of asthma severity, reflecting a more general asthma population compared to most previous studies. In a series of cohort analyses we estimated crude incidence rates of 11 potentially corticosteroid-related adverse events in users and non-users of OCS. We further conducted a series of case-control analyses nested within these cohorts to assess in more depth the association between timing, frequency, cumulative and average daily doses of OCS use, and the occurrence of these adverse events.

## Methods

### Study design and data source

We conducted a series of 11 cohort studies, each with a nested case-control analysis, using data from the CPRD.

The CPRD is a large and well-validated UK-based electronic primary care database with ongoing data collection, which contains anonymised, longitudinal data recorded by general practitioners (GPs) in daily clinical practice. Available information includes demographics, lifestyle factors, medical diagnoses, prescription history, specialist referrals, hospitalisations, and laboratory test results. The database encompasses approximately 10 million patient records from approximately 600 GP practices throughout the UK. Patients are representative of the UK population with regard to age, sex, and annual turnover rate [15]. Numerous studies have demonstrated the completeness and high validity of the recorded information [16, 17].

This study was approved by the Independent Scientific Advisory Committee for Medicines and Healthcare Products Regulatory Agency research (protocol number 16_060R).

### Study population

We identified all patients in the CPRD aged 18 years or older with incident or prevalent asthma (defined as requiring at least GINA step 2 treatment [4]) between January 2000 and December 2015. A qualifying asthma diagnosis was defined as a recorded Read code (clinical coding system used in UK primary care) for asthma at any time in the patient record, which was preceded and/or followed by an ICS prescription within 90 days prior or up to 365 days after. The date of cohort entry for these patients was either 1 January 2000 (for those who qualified before 1 January 2000, i.e. the prevalent asthma patients) or the date of the qualifying ICS prescription, whichever came last.

We excluded patients with (1.) < 3 years of recorded history in the CPRD before cohort entry, to allow for sufficient time to measure covariates (2.) a previous record of malignancy (except non-melanoma skin cancer), HIV/AIDS, alcoholism, or drug abuse, since these conditions and/or the associated drug therapies can lead to immune suppression and to a higher risk of several of the outcomes assessed, (3.) previously recorded differential diagnoses of asthma and/or a disease that is associated with chronic OCS use (rheumatoid arthritis, polymyalgia rheumatica, systemic lupus erythematosus, vasculitis, multiple sclerosis, chronic obstructive pulmonary disease, bronchiectasis, cystic fibrosis, interstitial pulmonary fibrosis, tuberculosis, autoimmune hepatitis, inflammatory bowel disease), (4.) ≥1 recorded OCS prescription within 2 years, or ≥4 recorded OCS prescriptions at any time, before cohort entry, to insure new use of OCS, (5.) prevalent asthma who did not have a recorded ICS prescription within 365 days before 1 January 2000, to ensure persisting asthma at cohort entry, (6.) the recorded outcome of interest (Table 1) prior to cohort entry.

**Table 1:** Definition of the outcomes of interest

Outcome	Definition
Bone-related conditions	Incident Read code of bone fracture (ankle, foot, toe, hand, finger, hip, femur, humerus, shoulder, clavicle, scapula, lower arm, elbow, wrist, vertebra or unspecific fracture) or osteoporosis.
Hypertension	Incident Read code of hypertension preceded or followed within 180 days prior until 180 days after by a prescription for an antihypertensive drug (ACE inhibitor, ARB, dihydropyridine CCB or thiazide diuretic). The first record of either hypertension or antihypertensive treatment was assigned the date of outcome occurrence.
Peptic ulcer	Incident Read code of peptic ulcer with or without haemorrhage.
Severe infections	Main analysis: incident Read code of an infection preceded or followed within 1 month by a record suggesting hospitalisation or i.v. anti-infective treatment. Sensitivity analysis restricted to patients with IHS linkage (conducted in the nested-case control study only): incident Read code of an infection preceded or followed within 1 month by a hospitalisation whose reason was infection (ICD-10 code of infection in the IHS data).
Herpes zoster	Incident Read code of herpes zoster
DM2	Incident Read code of DM2 and/or a first-time prescription for an oral antidiabetic drug, whichever came first. If a patient was prescribed insulin before any other antidiabetic drug during follow-up (and therefore likely was diagnosed with DM1), the event did not count as an outcome and the person was censored at the date of the insulin prescription.
Cataract	Incident Read code of cataract or a procedure for lens replacement, whichever came first.
Glaucoma	Incident Read code of glaucoma or a first-time prescription for intraocular pressure-lowering therapy, whichever came first.
CKD	Incident Read code of CKD or ≥2 recorded values of an eGFR of < 60 ml/min/1.73m^2^ within 365 days and separated by ≥90 days (calculated based on the CKD-EPI equation).
Affective disorders	Incident Read code of affective disorders (depression, bipolar affective disorder, manic episodes) preceded or followed within 180 days before or after by a prescription for an antidepressant or mood stabilizing drug, or a Read code indicating psychotherapy. The first record of either affective disorders or the respective treatment was assigned the date of outcome occurrence.
Cardiovascular events	Incident Read code of myocardial infarction, ischaemic heart disease, or ischaemic stroke.

Abbreviations: *ACE* angiotensin-converting-enzyme, *ARB* angiotensin II receptor blocker, *CCB* calcium channel blocker, *CKD* chronic kidney disease, *CKD-EPI* Chronic Kidney Disease Epidemiology Collaboration, *DM1* diabetes mellitus type 1, *DM2* diabetes mellitus type 2, *eGFR* estimated glomerular filtration rate, *IHS* integrated hospital episode statistics, *i.v.* intravenous

Read codes: thesaurus of clinical terms

### Cohort analyses

#### Follow-up and definition of outcomes

We followed patients from the date of cohort entry until the first record of an outcome of interest (Table 1), death, loss to follow-up, the end of the study period (30 December 2015), a code indicating the onset of an exclusion criterion outlined above, or the day after a period of 365 days without any recorded ICS prescriptions (presumed asthma remission). Most patients were included in all study cohorts, and right-censoring of follow-up was different for each outcome. If patients modified their prednisolone exposure as a consequence of the occurrence of an outcome, this could have affected the analyses of outcomes that occurred later on.

#### Person-time analyses and definition of exposure

We classified follow-up time for each patient into non-, current, or past use of OCS (subsequently referred to as oral prednisolone, since < 2% of the study population received OCS other than prednisolone). We defined non-use as the time period between cohort entry and the first oral prednisolone prescription or the censoring date, whichever came first. Current or past use was defined by estimating the supply of each prescription based on the total quantity of tablets prescribed and the dosage instructions, according to an algorithm developed based on a previous CPRD study [18] (see Additional file 1 for details on the algorithm, a scheme of the cohort analysis, and the extent of missing data on quantity of tablets or dosing instructions in each cohort).

### Nested case-control analyses

#### Definition of cases and controls

Cases were patients who had a first ever code for one of the study outcomes during the study period. The date of the recorded outcome will subsequently be referred to as the index date. We identified up to 4 controls for each case of the 11 study outcomes, from those in the asthma study population who did not have the outcome of interest prior to the case index date (risk set sampling). We matched controls to cases on index date, duration of follow-up (+/− 2 years), year of birth (+/− 2 years), sex, and duration of history in the database (+/− 0 years).

#### Definition of exposure

We defined non-users as patients with no oral prednisolone prescriptions prior to the index date, and ever users as patients with ≥1 oral prednisolone prescription recorded at any time before the index date. We further categorised ever users into current, recent, or past users, when their last prescription was recorded < 180, 180–365, or > 365 days before the index date, respectively.

We sub-classified current oral prednisolone users by (1.) cumulative dose prescribed before the index date (< 500 mg, 500-2000 mg, > 2000 mg), and (2.) average daily dose (≤1 mg, > 1 mg–5 mg, > 5 mg) ever, calculated based on the cumulative dose prescribed ever, divided by the days since the first prescription, and within periods of 1, 2, and 5 years before the index date, calculated based on the cumulative dose prescribed within 1, 2, or 5 years before the index date, divided by 365, 730, or 1825 days, respectively, and (3.) frequency of prescriptions before the index date (low use: on average 1 prescription/year; medium use: on average 2–3 prescriptions per year; high use: on average ≥ 4 prescriptions per year), was calculated based on the sum of prescriptions and the time since the first prescription.

#### Definition of covariates

We assessed the following potential confounders at any time before the index date of each case and control based on a priori clinical knowledge: alcohol consumption (most recent record before the index date: </≥14 units of alcohol per week, or unknown); smoking status (most recent record before the index date: non-, current-, ex-smoker, or unknown); body mass index (most recent record before the index date: < 18.5, 18.5–24.9, 25.0–29.9, ≥30.0, or unknown); current or past use (yes/no) of inhaled bronchodilators, nonsteroidal anti-inflammatory drugs, platelet aggregation inhibitors, anticoagulants, proton pump inhibitors, vitamin D/calcium, bisphosphonates, and immunosuppressants; the number of ICS prescriptions (as a continuous variable), and the Charlson Comorbidity Index (a summary measure for disease burden of a patient [19]; as a continuous variable, and categorized in 0, 1–3, 4–6, > 6).

### Statistical analysis

#### Cohort analyses

We performed a series of 11 cohort studies to assess crude incidence rates (IRs) with 95% confidence intervals (CIs) for each outcome among non-users, ever users (current and past users combined), and current users of oral prednisolone. We compared IRs for ever- and current users with those for non-users by calculating crude incidence rate ratios (IRRs) with 95% CIs.

#### Nested case-control analyses

We conducted a series of 11 nested case-control analyses for each outcome of interest to assess in more detail the association between oral prednisolone use and the identified adverse events. We compared different oral prednisolone treatment patterns between cases and controls by calculating odds ratios (ORs) with 95% CIs using conditional logistic regression models. To control for confounding, we computed multivariable odds ratios (ORs_adj_) adjusting for the potential confounding variables defined above.

#### Sensitivity analysis

We conducted a sensitivity analysis to assess whether IRs, IRRs, and ORs (by current, recent, and past use of prednisolone), change according to whether patients were included in the cohorts at January 01 2000 (prevalent asthma patients) or thereafter (incident asthma patients).

We conducted all analyses using SAS 9.4 software (SAS Institute, Cary, NC), and we defined statistical significance at the alpha-level of 0.05.

## Results

### Characteristics of the study population

Between 165,900 and 269,368 asthma patients were included in each of the 11 cohorts, of whom between 836 and 16,192 developed an outcome of interest. The large majority of case patients could be matched to at least 1 control for the nested case-control analyses. The mean age of cases ranged from 40.8 years for affective disorders to 72.7 years for cataract. Females were more frequent among cases of all examined outcomes (Table 2).

**Table 2:** Characteristics of the cohorts and cases for each of the 11 study outcomes

	Bone-related conditions	Hypertension	Peptic ulcer	Severe infections	Herpes zoster	DM2	Cataract	Glaucoma	CKD	Affective disorders	Cardiovascular events
Cohort analysis											
Individuals at cohort entry, n	219,174	223,940	267,569	244,207	262,847	262,304	265,964	269,368	267,138	165,900	260,995
Individuals with cohort entry on 1 January 2000, n	40,484	37,250	46,439	44,621	46,016	46,046	46,324	46,892	47,549	30,722	44,549
Individuals with cohort entry after 1 January 2000, n	178,690	186,690	221,130	199,586	216,831	216,258	219,640	222,476	219,589	135,178	216,446
Cases, n	8927	8162	836	16,192	4175	5195	5356	1473	8825	4965	4028
Total follow-up time, py	682,398	653,565	850,874	744,410	824,689	820,055	830,289	854,208	825,108	525,577	816,959
Nested case-control analysis											
Cases^a^, n	8907	8148	835	16 160^b^ 2748^c^	4163	5189	5327	1467	8778	4962	4014
Controls, n	35,445	32,431	3328	64,316^b^ 10839^c^	16,605	20,715	21,019	5832	34,762	19,809	15,945
Mean age of cases, years (SD)	54.3 (+/− 19.6)	58.9 (+/− 12.9)	63.1 (+/− 16.7)	51.8 (+/− 19.7)^b^ 58.6 (+/− 20.8)^c^	57.3 (+/− 17.5)	58.1 (+/− 15.3)	72.7 (+/− 10.9)	68.0 (+/− 13.0)	71.3 (+/− 12.0)	40.8 (+/− 16.7)	67.4 (+/− 12.4)
Females among cases, %	69.1	57.4	60.2	67.7^b^ 64.6^c^	66.0	60.1	68.9	62.9	73.1	62.2	53.0

Abbreviations: *CKD* chronic kidney disease, *DM2* diabetes mellitus type 2, *py* person-years, *SD* standard deviation

^a^Cases from the cohort analysis that could be matched to at least 1 control on the index date, duration of follow-up (+/− 2 years), year of birth (+/− 2 years), sex, and duration of history on the database (+/− 0 years)

^b^Main analysis

^c^Sensitivity analysis restricted to patients with integrated hospital episode statistics linkage

A study flowchart and detailed characteristics of cases and controls are provided in Additional file 2.

### Cohort analyses

Compared with non-users, current users of oral prednisolone had statistically significantly increased crude IRRs for all outcomes except glaucoma. The risk differences between current and non-users of oral prednisolone were greatest for severe infections (60.2/1000 person-years), bone-related conditions (6.2/1000 person-years), and chronic kidney disease (CKD, 5.5/1000 person-years). The crude IRRs for ever users of oral prednisolone tended to be lower than the IRRs for current users (reference: non-users), but remained statistically significantly increased for most outcomes. For some outcomes the IRRs were only minimally increased and effects could be due to bias or confounding (Table 3).

**Table 3:** Crude incidence rates and incidence rate ratios of 11 study outcomes among asthma patients, stratified by non-use, ever use, and current use of oral prednisolone

Use of oral prednisolone	Bone-related conditions	Hypertension	Peptic ulcer	Severe infections	Herpes zoster	DM2	Cataract	Glaucoma	CKD	Affective disorders	Cardiovascular events
Non-use (reference)											
Cases, n	6174	5867	591	10,115	2882	3534	3639	1057	6034	3602	2868
Person-years	509,914	493,646	631,164	567,254	614,128	609,903	618,128	633,404	616,192	402,782	607,320
IR/ 1000 person-years (95% CI)	12.1 (11.8–12.4)	11.9 (11.6–12.2)	0.9 (0.9–1.0)	17.8 (17.5–18.2)	4.7 (4.5–4.9)	5.8 (5.6–6.0)	5.9 (5.7–6.1)	1.7 (1.6–1.8)	9.8 (9.5–10.0)	8.9 (8.7–9.2)	4.7 (4.5–4.9)
Ever use											
Cases, n	2753	2295	245	6077	1293	1661	1717	416	2791	1363	1160
Person-years	172,484	159,919	219,711	177,156	210,560	210,152	212,161	220,804	208,916	122,796	209,638
IR/1000 person-years (95% CI)	16.0 (15.4–16.6)	14.4 (13.8–14.9)	1.1 (1.0–1.3)	34.3 (33.4–35.2)	6.1 (5.8–6.5)	7.9 (7.5–8.3)	8.1 (7.7–8.5)	1.9 (1.7–2.1)	13.4 (12.9–13.9)	11.1 (10.5–11.7)	5.5 (5.2–5.9)
IRR (95% CI)	1.3 (1.3–1.4)	1.2 (1.2–1.3)	1.2 (1.0–1.4)	1.9 (1.9–2.0)	1.3 (1.2–1.4)	1.4 (1.3–1.4)	1.4 (1.3–1.5)	1.1 (1.0–1.3)	1.4 (1.3–1.4)	1.2 (1.2–1.3)	1.2 (1.1–1.3)
Current use											
Cases, n	533	451	51	2268	257	398	272	62	541	269	236
Person-years	29,141	27,032	36,864	29,074	35,586	35,468	35,723	37,125	35,364	19,803	35,269
IR/1000 person-years (95% CI)	18.3 (16.7–19.8)	16.7 (15.1–18.2)	1.4 (1.0–1.8)	78.0 (74.8–81.2)	7.2 (6.3–8.1)	11.2 (10.1–12.3)	7.6 (6.7–8.5)	1.7 (1.3–2.1)	15.3 (14.0–16.6)	13.6 (12.0–15.2)	6.7 (5.8–7.5)
IRR (95% CI)	1.5 (1.4–1.6)	1.4 (1.3–1.5)	1.5 (1.1–2.0)	4.4 (4.2–4.6)	1.5 (1.4–1.7)	1.9 (1.7–2.2)	1.3 (1.1–1.5)	1.0 (0.8–1.3)	1.6 (1.4–1.7)	1.5 (1.3–1.7)	1.4 (1.2–1.6)

Abbreviations: *CI* confidence interval, *CKD* chronic kidney disease, *DM2* diabetes mellitus type 2, *IR* incidence rate, *IRR* incidence rate ratio

### Nested case-control analyses

After adjusting for potential confounding, current users of oral prednisolone had statistically significantly increased ORs of all investigated outcomes except ocular diseases and hypertension, as compared with non-users (Table 4).

**Table 4:** Odds ratios for the 11 study outcomes in relation to oral prednisolone use among asthma patients, by current, recent or past use

Use of oral prednisolone	Bone-related conditions	Hypertension	Peptic ulcer	Severe Infections^a^	Herpes zoster	DM2	Cataract	Glaucoma	CKD	Affective disorders	Cardiovascular events
No use (reference)											
Cases, n (%)	4558 (51.2)	4397 (54.0)	416 (49.8)	7642 (47.3)	2056 (49.4)	2571 (49.6)	2582 (48.5)	769 (52.4)	4344 (49.5)	2769 (55.8)	2096 (52.2)
Controls, n (%)	19,038 (53.7)	17,802 (54.9)	1805 (54.2)	36,039 (56.0)	8875 (53.5)	11,071 (53.4)	10,641 (50.6)	3048 (52.3)	17,768 (51.1)	12,016 (60.7)	8827 (55.4)
Current use (< 180 d before ID)											
Cases, n (%)	846 (9.5)	654 (8.0)	80 (9.6)	2227 (13.8)	406 (9.8)	596 (11.5)	433 (8.1)	119 (8.1)	811 (9.2)	434 (8.8)	379 (9.4)
Controls, n (%)	2614 (7.4)	2414 (7.4)	225 (6.8)	4489 (7.0)	1307 (7.9)	1617 (7.8)	1612 (7.7)	463 (7.9)	2763 (8.0)	1210 (6.1)	1138 (7.1)
OR crude (95% CI)	1.36 (1.26–1.47)	1.10 (1.01–1.20)	1.57 (1.23–2.01)	2.38 (2.26–2.50)	1.36 (1.22–1.52)	1.60 (1.46–1.75)	1.11 (1.00–1.23)	1.02 (0.84–1.24)	1.21 (1.12–1.30)	1.58 (1.42–1.75)	1.41 (1.26–1.58)
OR_adj_^b^ (95% CI)	1.27 (1.17–1.37)	1.03 (0.94–1.12)	1.47 (1.12–1.92)	2.16 (2.05–2.27)	1.32 (1.19–1.48)	1.35 (1.22–1.49)	1.03 (0.93–1.15)	1.06 (0.87–1.29)	1.14 (1.06–1.24)	1.47 (1.32–1.63)	1.33 (1.18–1.49)
Recent use (180–365 d before ID)											
Cases, n (%)	519 (5.8)	405 (5.0)	50 (6.0)	1121 (6.9)	267 (6.4)	293 (5.7)	310 (5.8)	75 (5.1)	478 (5.5)	308 (6.2)	212 (5.3)
Controls, n (%)	1858 (5.2)	1608 (5.0)	159 (4.8)	3087 (4.8)	889 (5.4)	1036 (5.0)	1102 (5.2)	297 (5.1)	1850 (5.3)	871 (4.4)	765 (4.8)
OR crude (95% CI)	1.18 (1.08–1.29)	1.02 (0.92–1.13)	1.36 (1.01–1.83)	1.74 (1.63–1.86)	1.32 (1.16–1.50)	1.23 (1.09–1.39)	1.17 (1.04–1.31)	0.99 (0.75–1.26)	1.06 (0.97–1.17)	1.55 (1.38–1.75)	1.18 (1.03–1.37)
OR_adj_^b^ (95% CI)	1.12 (1.02–1.24)	0.95 (0.85–1.05)	1.19 (0.87–1.62)	1.59 (1.49–1.70)	1.29 (1.13–1.47)	1.05 (0.92–1.19)	1.12 (0.99–1.26)	1.00 (0.79–1.28)	1.00 (0.91–1.10)	1.49 (1.32–1.69)	1.11 (0.95–1.29)
Past use (> 365 d before ID)											
Cases, n (%)	2984 (33.5)	2692 (33.0)	289 (34.6)	5170 (32.0	1434 (34.5)	1729 (33.3)	2002 (37.6)	504 (34.4)	3145 (35.8)	1451 (29.2)	1327 (33.1)
Controls, n (%)	11,935 (33.7)	10,607 (32.7)	1139 (34.2)	20,701 (32.2)	5534 (33.3)	6991 (33.8)	7664 (36.5)	2024 (34.7)	12,381 (35.6)	5712 (28.8)	5215 (32.7)
OR crude (95% CI)	1.05 (1.00–1.10)	1.03 (0.98–1.08)	1.11 (0.95–1.30)	1.20 (1.16–1.25)	1.13 (1.05–1.21)	1.08 (1.01–1.15)	1.08 (1.02–1.15)	0.99 (0.88–1.11)	1.04 (0.99–1.09)	1.12 (1.05–1.19)	1.08 (1.01–1.16)
OR_adj_^b^ (95% CI)	1.03 (0.98–1.08)	0.97 (0.92–1.02)	1.05 (0.89–1.25)	1.14 (1.10–1.19)	1.13 (1.05–1.21)	0.98 (0.92–1.05)	1.04 (0.98–1.10)	1.00 (0.89–1.13)	1.01 (0.97–1.07)	1.10 (1.02–1.18)	1.06 (0.98–1.14)

Abbreviations: *CI* confidence interval, *CKD* chronic kidney disease, *d* days, *DM2* diabetes mellitus type 2, *ID* index date, *OR*_*adj*_ adjusted odds ratio

^a^Results from main analysis (the sensitivity analysis restricted to patients with integrated hospital episode statistics yielded similar results, data not shown)

^b^Adjusted for alcohol consumption, smoking status, body mass index, current or past use of inhaled bronchodilators, nonsteroidal anti-inflammatory drugs, platelet aggregation inhibitors, anticoagulants, proton pump inhibitors, vitamin D/calcium, bisphosphonates, immunosuppressants, number of prescriptions of inhaled corticosteroids, and Charlson Comorbidity Index

**Fig. 1:**
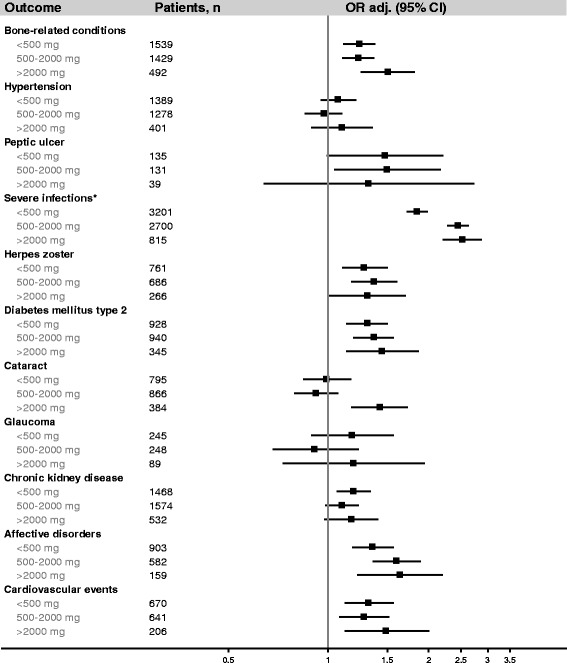
Current use of oral prednisolone and the risk of 11 study outcomes among asthma patients, by cumulative dose ever prescribed before the index date**.** Abbreviations: OR_adj_, adjusted odds ratio; CI, confidence interval. *Results from main analysis (the sensitivity analysis restricted to patients with integrated hospital episode statistics yielded similar results, data not shown)**.** Odds ratios are presented on a logarithmic scale and were adjusted for alcohol consumption, smoking status, body mass index, current or past use of inhaled bronchodilators, nonsteroidal anti-inflammatory drugs, platelet aggregation inhibitors, anticoagulants, proton pump inhibitors, vitamin D/calcium, bisphosphonates, immunosuppressants, number of prescriptions of inhaled corticosteroids (as a continuous variable), and Charlson Comorbidity Index (as a continuous variable)

**Fig. 2:**
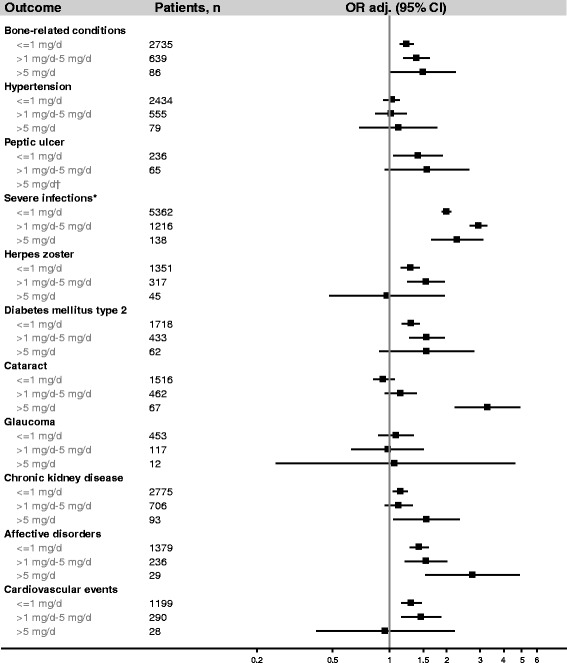
Current use of oral prednisolone and the risk of 11 study outcomes among asthma patients, by the average daily dose prescribed within 2 years before the index date**.** Abbreviations: OR_adj_, adjusted odds ratio; CI, confidence interval. ^**†**^ Cells contain < 5 patients, owing to ethics regulations to preserve confidentiality, odds ratios are not displayed. *Results from main analysis (the sensitivity analysis restricted to patients with integrated hospital episode statistics yielded similar results, data not shown). Odds ratios are presented on a logarithmic scale and were adjusted for alcohol consumption, smoking status, body mass index, current or past use of inhaled bronchodilators, nonsteroidal anti-inflammatory drugs, platelet aggregation inhibitors, anticoagulants, proton pump inhibitors, vitamin D/calcium, bisphosphonates, immunosuppressants, number of prescriptions of inhaled corticosteroids (as a continuous variable), and Charlson Comorbidity Index (as a continuous variable)

**Fig. 3:**
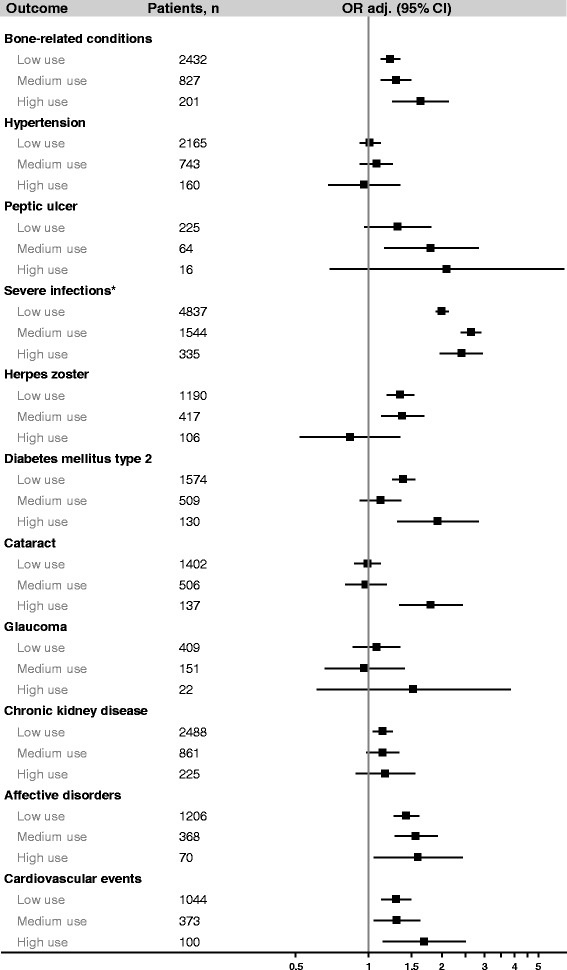
Current use of oral prednisolone and the risk of 11 study outcomes among asthma patients, by frequency of use before the index date (low use: on average 1 prescription/year; medium use: on average 2–3 prescriptions/year; high use: on average ≥ 4 prescriptions/year). Abbreviations: OR_adj_, adjusted odds ratio; CI, confidence interval. *Results from main analysis (the sensitivity analysis restricted to patients with integrated hospital episode statistics yielded similar results, data not shown). Odds ratios are presented on a logarithmic scale and were adjusted for alcohol consumption, smoking status, body mass index, current or past use of inhaled bronchodilators, nonsteroidal anti-inflammatory drugs, platelet aggregation inhibitors, anticoagulants, proton pump inhibitors, vitamin D/calcium, bisphosphonates, immunosuppressants, number of prescriptions of inhaled corticosteroids (as a continuous variable), and Charlson Comorbidity Index (as a continuous variable)

Severe infection was the only outcome with a substantially increased OR (2.16). Peptic ulcer (OR = 1.47) and affective disorders (OR = 1.47) had smaller increases in risk, and several other outcomes had marginally increased risks (herpes zoster, cardiovascular events, diabetes mellitus type 2 [DM2], and bone related conditions). Cataract was associated with an increase in risk at cumulative doses > 2000 mg, average daily doses > 5 mg/day, or ≥4 prescriptions/year (ORs = 1.43, 3.29, and 1.80, respectively), but not at lower doses (Figs. 1, 2 and 3). Hypertension, glaucoma, and CKD were not associated with current use of oral prednisolone (Table 4). Recent users had small increases in risk of severe infections, affective disorders, and herpes zoster, but not of other outcomes. There were no elevated risks among past users (Table 4).

Figures 1, 2 and 3 display the results of the sub-analyses among current users of oral prednisolone by cumulative dose before the index date, average daily dose within 2 years before the index date (the figures for the average daily dose prescribed ever, within 1 year, or within 5 years before the index date pointed in the same direction and are not shown), and frequency of use. Possible dose response-relationships were observed for bone-related conditions, severe infections, cataract, affective disorders, DM2, and cardiovascular events. For herpes zoster and peptic ulcer, no dose-response relationships were observed.

The results of the sensitivity analysis were not considerably different between prevalent or incident asthma patients (data not shown).

## Discussion

In this large observational study based on CPRD data, oral prednisolone use was associated with the occurrence of various incident diseases in adult patients with asthma. This association was strongest in current users of oral prednisolone, and gradually declined in recent or past users, compared with non-users. There was a strong association with current use of oral prednisolone and severe infections and possible dose response relationships with bone-related conditions, cataract, DM2, affective disorders, and cardiovascular events. There was also a small non-dose dependent association with herpes zoster and peptic ulcer. In contrast, oral prednisolone use was not associated with the risk of glaucoma, CKD, or hypertension, irrespective of timing of use or the prescribed dose.

The ORs of all outcomes associated with current oral prednisolone use, except cataract, were also increased in patients who had only received an average of 1 prescription per year, a cumulative dose < 500 mg, and/or an average daily dose ≤1 mg within 2 years before the index date. As we did not observe increased ORs in patients with past use of these intermittent and low dose regimens, mere confounding by generally poorer health of prednisolone users compared with non-users is unlikely. Furthermore, cataract, a slowly progressive disease which is associated with frailty and overall disease burden [20], was only associated with the highest level of exposure for all categories of use: highest cumulative and daily dose and most frequent oral prednisolone regimens.

Previous studies that assessed OCS-related adverse events among asthma patients found similarly increased risk estimates of bone-related, gastrointestinal, infectious, metabolic, renal, psychiatric, and cardiovascular complications in association with OCS use [10–14]. In accordance with our results, previous studies did not find an association between OCS exposure and the occurrence of glaucoma in asthma patients [10, 11, 14] which is also consistent with findings from studies of non-asthmatic OCS users [21, 22]. As previously demonstrated, the risk of cataract in association with OCS use appears to increase notably after long treatment duration or at high cumulative doses or frequency of use [14, 23, 24].

Previous studies among asthma patients found a statistically significant association between OCS use and hypertension [10–12, 14] while we observed no such association after adjusting for confounding. This discrepancy might be explained by our stricter definition of hypertension which required patients to receive antihypertensive treatment in addition to a hypertension diagnosis, whereas in all other studies, patients only needed to have a hypertension diagnosis.

Although dose-response relationships between OCS use and various adverse events have been previously reported [6, 7, 12, 23–25], some of which among US populations of patients with asthma [12–14], this comprehensive longitudinal study adds evidence from the UK, based on one of the largest and best validated medical records database worldwide. This is the first study providing absolute and relative frequencies of potential corticosteroid-related complications among patients with asthma who used or did not use OCS, whereas previous studies reported relative frequencies only [12–14]. While previous cohort studies either classified OCS exposure based on cumulative dose [12, 13] or on the frequency of OCS prescriptions issued per year [14], the results of our study provide evidence that no matter which of these exposure classification approaches is taken, observed associations between the intensity of OCS use and related adverse events remain stable.

Despite these strengths, there are limitations to our study. As in all electronic database studies, our prednisolone exposure definition was based on issued prescriptions and not on actual medication intake. The calculated cumulative dose represents the cumulative dose ever prescribed rather than ingested OCS; e.g. the observed associations between oral prednisolone use and investigated outcomes were more likely to be under- rather than overestimated. Additionally, we had some missing data on quantity of tablets and dosage instructions necessary to calculate current and past use periods in the cohort studies. Although diagnoses are well validated in the CPRD, we cannot rule out the possibility of coding errors. To avoid including false positive outcomes, we based our definitions of most outcomes not only on Read codes, but also on recorded prescriptions or procedures for these specific disorders. However, missing diagnoses could have been an issue particularly for the outcomes peptic ulcer (especially types without haemorrhage) or bone-related conditions (especially osteoporosis without bone-fractures), as these outcomes may have gone unnoticed by the GP. This type of misclassification could have biased our results towards the null hypothesis if it was not differential across users and non-users of prednisolone, or, if users of prednisolone were more closely observed with regard to these outcomes, the risk estimates could have been overestimated. While we could distinguish between more or less frequent average use of oral prednisolone, we could not classify patients into continuous or burst users, as available dosage instructions were not detailed enough. Furthermore, it was not possible to disentangle potential effects of oral prednisolone from an underlying effect of asthma severity, as more severe asthma patients by definition are more likely to be prescribed oral prednisolone. Previous studies have demonstrated that asthma patients have a higher prevalence of metabolic, cardiovascular, and psychiatric diseases, compared with the general population [26–28]. These associations are more likely to be bi- than unidirectional, and supposedly based on common pathophysiological parameters of these diseases [26–28]. We are however not aware of studies reporting a higher prevalence of these comorbidities in patients with severe asthma compared with less severe asthma, and confounding by asthma severity for these outcomes was unlikely in this study, as we observed no association between current oral prednisolone use and hypertension, or between past use of oral prednisolone and cardiovascular events, DM2, or affective disorders. We did not adjust our analyses for multiple comparisons because all analyses were planned in advance and based on an a priori hypothesis [29, 30].

## Conclusion

In conclusion, this study adds evidence from the UK that new use of oral prednisolone is associated with an increased risk of various incident diseases. Some outcomes were associated with not only high dose and frequent oral prednisolone use, but also with low dose and intermittent regimens. A possible dose-response relationship was observed for bone-related conditions, severe infections, cataract, affective disorders, DM2, and cardiovascular events. Our results support the need of developing new treatments for severe asthma with a better safety profile than that of OCS.

## Additional files

Additional file 1: Algorithm used to calculate current and past use in the cohort analysis. Schematic description of the cohort analysis. Overview on the extent of missing data to calculate current use exposure in each cohort. (PDF 327 kb)

Additional file 2: Flowchart of the study population. Detailed information on characteristics and co-medication of cases and controls for each outcome at the index date. (PDF 896 kb)
