# Supplementary material for: Adverse events profile of oral corticosteroids among asthma patients in the UK: cohort study with a nested case-control analysis
Source: Respir Res. 2018 Apr 27;19:75. doi: 10.1186/s12931-018-0742-y (PMC5921395; doi:10.1186/s12931-018-0742-y)
Supplement: Supplementary file 1 — Algorithm used to calculate current and past use in the cohort analysis. Schematic description of the cohort analysis. Overview on the extent of missing data to calculate current use exposure in each cohort. (PDF 327 kb) [file 12931_2018_742_MOESM1_ESM.pdf]

Additional file 1

Algorithm used to calculate current and past use in the cohort analysis:

A current use period started on the day of a recorded oral prednisolone prescription and continued through the last day of the prescription supply plus 90 days. If a new oral prednisolone prescription was recorded prior to the last day of the estimated supply of the previous prescription, we added the estimated supply of the new prescription to the previous supply length with a maximum overlap period of supply of the two prescriptions of 180 days (i.e. the maximum exposed period to current use without an intermittently issued prescription was 180 days). A past use period started after the end of a current use period (prescription supply plus 90 days) and lasted until a new prescription was issued or the patient was censored. When data on quantity of tablets or dosage instructions was missing, we estimated the prescription duration based on the median prescription length among patients of the same age and sex.

Schematic description of the cohort analysis:

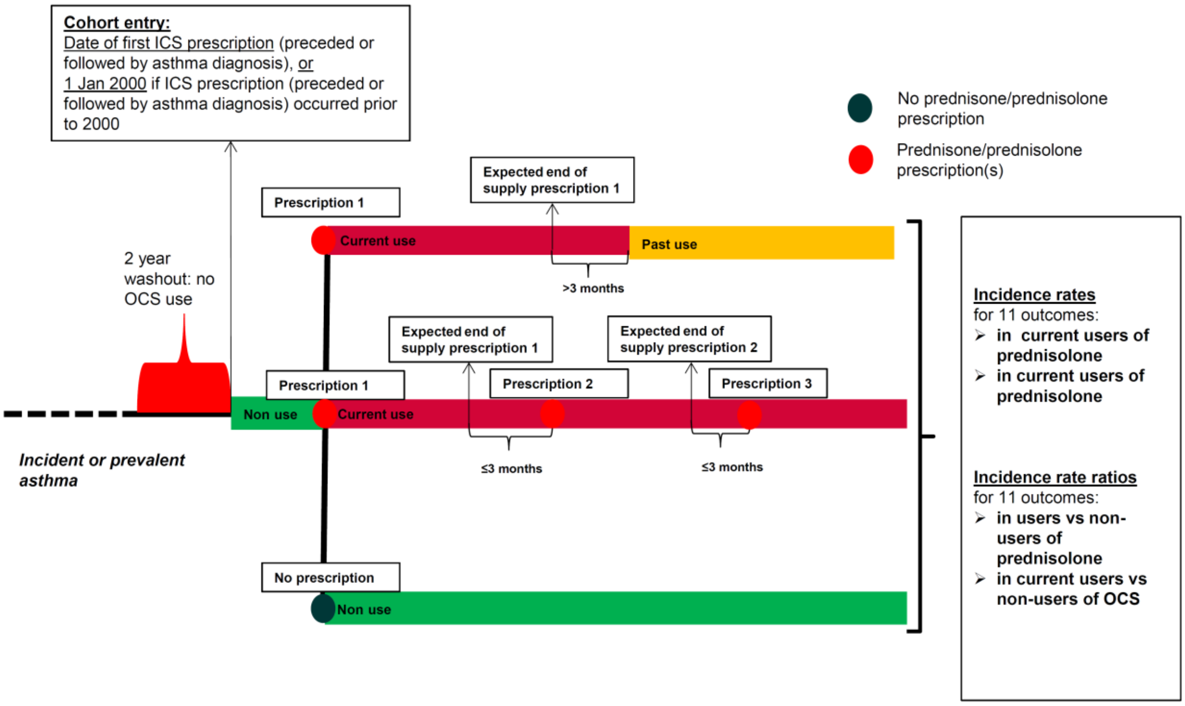

Overview on the extent of missing data to calculate current use exposure in each cohort:

Outcome	Patients with current use period during follow-up with complete data to calculate current use exposure windows, n (%)	Patients with current use period during follow-up for whom data was imputed to calculate current use exposure window, n (%)
Bone-related conditions	32 117 (61.8)	19 888 (38.2)
Osteoporosis	32 651 (61.5)	20 470 (38.5)
Hypertension	30 938 (62.1)	18 921 (37.9)
Ulcer	39 686 (61.5)	24 803 (38.5)
Severe infections	34 788 (62.4)	21 004 (37.6)
Herpes zoster	38 659 (61.6)	24 080 (38.4)
Type 2 diabetes mellitus	38 683 (61.7)	24 009 (38.3)
Cataract	39 116 (61.7)	24 249 (38.3)
Glaucoma	39 993 (61.6)	24 937 (38.4)
Chronic kidney disease	39 059 (61.8)	24 108 (38.2)
Affective disorders	22 055 (61.3)	13 948 (38.7)
Cardiovascular disease	38 267 (61.6)	23 814 (38.4)
