# Supplementary material for: Adverse events profile of oral corticosteroids among asthma patients in the UK: cohort study with a nested case-control analysis
Source: Respir Res. 2018 Apr 27;19:75. doi: 10.1186/s12931-018-0742-y (PMC5921395; doi:10.1186/s12931-018-0742-y)
Supplement: Supplementary file 2 — Flowchart of the study population. Detailed information on characteristics and co-medication of cases and controls for each outcome at the index date. (PDF 896 kb) [file 12931_2018_742_MOESM2_ESM.pdf]

# Additional file 2

**Flowchart study population:**

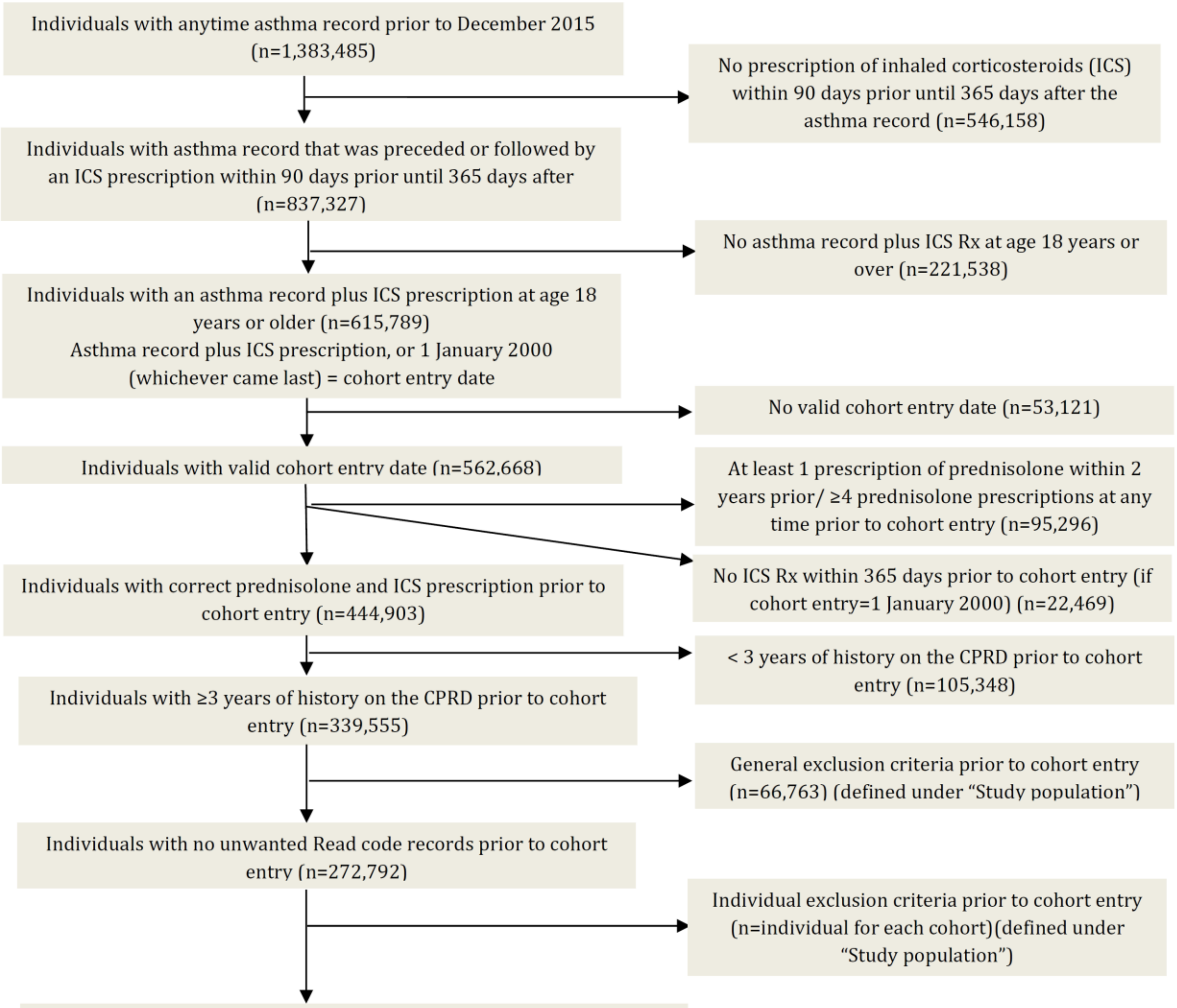


| | **No. of individuals at cohort entry** | **No. of cases during follow-up** |
|---|---|---|
| Bone-related conditions | 219,174 | 8,927 |
| Hypertension | 223,940 | 8,162 |
| Ulcer | 267,569 | 836 |
| Severe infections | 244,207 | 16,192 |
| Herpes zoster | 262,847 | 4,175 |
| Diabetes | 262,304 | 5,195 |
| Cataract | 265,964 | 5,356 |
| Glaucoma | 269,368 | 1,473 |
| Chronic kidney disease | 267,138 | 8,825 |
| Psychiatric events | 165,900 | 4,965 |
| Cardiovascular disease | 260.995 | 4.028 |

**Detailed information on characteristics and co-medication of cases and controls for each outcome at the index date:**

Characteristics and co-medication of cases with **bone-related conditions** and matched controls at the index date

| | No. of Cases (%) (N=8,907) | No. of Controls (%) (N=35,445) | Crude OR | 95% CI |
|---|---|---|---|---|
| Male | 2,752 (30.9) | 10,956 (30.9) | NA | NA |
| Female | 6,155 (69.1) | 24,489 (69.1) | NA | NA |
| **Age** | | | | |
| 18-39 | 2,321 (26.1) | 9,263 (26.1) | NA | NA |
| 40-59 | 2,713 (30.5) | 10,858 (30.6) | NA | NA |
| 60-79 | 2,947 (33.1) | 11,781 (33.2) | NA | NA |
| ≥80 | 926 (10.4) | 3,543 (10.0) | NA | NA |
| **Smoking status** | | | | |
| non | 4,495 (50.5) | 18,672 (52.7) | 1.00 | reference |
| current | 1,438 (16.1) | 5,020 (14.2) | 1.20 | 1.12-1.29 |
| ex | 2,767 (31.1) | 10,935 (30.9) | 1.05 | 0.99-1.10 |
| unknown | 207 (2.3) | 818 (2.3) | 1.06 | 0.90-1.25 |
| **Alcohol consumption (average units per week)** | | | | |
| none | 4,231 (47.5) | 16,714 (47.2) | 1.00 | reference |
| <14 | 2,786 (31.3) | 11,351 (32.0) | 0.98 | 0.92-1.03 |
| ≥14 | 862 (9.7) | 3,150 (8.9) | 1.09 | 1.00-1.19 |
| unknown | 1,028 (11.5) | 4,230 (11.9) | 0.96 | 0.88-1.04 |
| **Body mass index, kg/m$^2$** | | | | |
| <18.5 | 197 (2.2) | 612 (1.7) | 1.17 | 0.99-1.38 |
| 18.5-24.9 | 3,006 (33.8) | 10,850 (30.6) | 1.00 | reference |
| 25-29.9 | 2,633 (29.6) | 10,971 (31.0) | 0.86 | 0.81-0.91 |
| ≥30 | 2,138 (24.0) | 9,327 (26.3) | 0.82 | 0.77-0.87 |
| unknown | 933 (10.5) | 3,685 (10.4) | 0.93 | 0.85-1.02 |
| **Concomitant[a] drug use** | | | | |
| SABA | 7,646 (85.8) | 29,977 (84.6) | 1.10 | 0.92-1.32 |
| SAMA | 331 (3.7) | 1,154 (3.3) | 1.17 | 1.03-1.32 |
| ICS (mono preparations) | 6,353 (71.3) | 25,892 (73.1) | 0.96 | 0.86-1.08 |
| ICS+LABA | 3,048 (34.2) | 11,350 (32.0) | 1.13 | 1.07-1.20 |
| Asthma add-on therapy[b] | 420 (4.7) | 1,511 (4.3) | 1.13 | 1.01-1.26 |
| Platelet aggregation inhibitors | 1,226 (13.8) | 4,343 (12.3) | 1.18 | 1.09-1.27 |
| Anticoagulants | 862 (2.4) | 239 (2.7) | 1.10 | 0.95-1.27 |
| Proton pump inhibitors | 2,042 (22.9) | 6,769 (19.1) | 1.34 | 1.26-1.43 |
| Immunosuppressants[c] | 25 (0.3) | 69 (0.2) | 1.45 | 0.92-2.30 |
| Bisphosphonates | 231 (2.6) | 287 (0.8) | 3.30 | 2.76-3.94 |
| Vitamin D | 468 (5.3) | 1,030 (2.9) | 1.90 | 1.69-2.13 |
| **Charlson Comorbidity Index** | | | | |
| Median (25% perc./75% perc./range) | 1 (1/1/8) | 1 (1/1/10) | NA | NA |
| **Charlson Comorbidity Index (categories)** | | | | |
| 0 | 161 (1.8) | 745 (2.1) | 1.00 | reference |
| 1-3 | 8,369 (94.0) | 33,443 (94.4) | 1.18 | 1.01-1.37 |
| 4-6 | 349 (3.9) | 1,185 (3.3) | 1.38 | 1.14-1.67 |
| 7-10 | 28 (0.3) | 72 (0.2) | 1.80 | 1.18-2.74 |
| **Number of prescriptions ICS prior** | | | | |
| Median (25% perc./75% perc./range) | 30 (12/58/383) | 29 (12/56/361) | NA | NA |

Characteristics and co-medication of cases with **hypertension** and matched controls at the index date

| | No. of Cases (%) (N=8,148) | No. of Controls (%) (N=32,431) | Crude OR | 95% CI |
|---|---|---|---|---|
| Male | 3,469 (42.6) | 13,790 (42.5) | NA | NA |
| Female | 4,679 (57.4) | 18,641 (57.5) | NA | NA |
| **Age** | | | | |
| 18-39 | 533 (6.5) | 2,138 (6.6) | NA | NA |
| 40-59 | 3,607 (44.3) | 14,400 (44.4) | NA | NA |
| 60-79 | 3,582 (44.0) | 14,316 (44.1) | NA | NA |
| ≥80 | 426 (5.2) | 1,577 (4.9) | NA | NA |
| **Smoking status** | | | | |
| non | 3,966 (48.7) | 16,377 (50.5) | 1 | reference |
| current | 994 (12.2) | 4,265 (13.1) | 0.96 | 0.89-1.04 |
| ex | 3,015 (37.0) | 10,923 (33.7) | 1.15 | 1.08-1.21 |
| unknown | 173 (2.1) | 866 (2.7) | 0.81 | 0.68-0.96 |
| **Alcohol consumption (average units per week)** | | | | |
| none | 3,766 (46.2) | 13,661 (42.1) | 1 | reference |
| <14 | 2,750 (33.8) | 11,578 (35.7) | 0.86 | 0.82-0.91 |
| ≥14 | 1,171 (14.4) | 4,026 (12.4) | 1.06 | 0.98-1.14 |
| unknown | 461 (5.7) | 3,166 (9.8) | 0.53 | 0.47-0.58 |
| **Body mass index, kg/m$^2$** | | | | |
| <18.5 | 51 (0.6) | 360 (1.1) | 0.87 | 0.65-1.17 |
| 18.5-24.9 | 1,754 (21.5) | 10,967 (33.8) | 1 | reference |
| 25-29.9 | 2,844 (34.9) | 11,424 (35.2) | 1.57 | 1.47-1.68 |
| ≥30 | 2,970 (36.5) | 6,635 (20.5) | 2.92 | 2.72-3.12 |
| unknown | 529 (6.5) | 3,045 (9.4) | 1.05 | 0.95-1.17 |
| **Concomitant[a] drug use** | | | | |
| SABA | 6,832 (83.9) | 26,526 (81.8) | 1.18 | 1.00-0.39 |
| SAMA | 312 (3.8) | 1,181 (3.6) | 1.06 | 0.93-1.21 |
| ICS (mono preparations) | 6,140 (75.4) | 24,504 (75.6) | 1.08 | 0.95-1.23 |
| ICS+LABA | 2,469 (30.3) | 9,604 (29.6) | 1.04 | 0.98-1.11 |
| Asthma add-on therapy[b] | 346 (4.3) | 1,304 (4.0) | 1.06 | 0.94-1.20 |
| Platelet aggregation inhibitors | 723 (8.9) | 2,297 (7.1) | 1.31 | 1.19-1.43 |
| Anticoagulants | 110 (1.4) | 474 (1.5) | 0.91 | 0.74-1.13 |
| Proton pump inhibitors | 1,714 (21.0) | 5,681 (17.5) | 1.27 | 1.20-1.36 |
| Immunosuppressants[c] | 15 (0.2) | 80 (0.3) | 0.74 | 0.43-1.29 |
| Bisphosphonates | 188 (2.3) | 911 (2.8) | 0.80 | 0.68-0.94 |
| Vitamin D | 287 (3.5) | 1,168 (3.6) | 0.96 | 0.84-1.10 |
| **Charlson Comorbidity Index** | | | | |
| Median (25% perc./75% perc./range) | 1 (1/1/8) | 1 (1/1/7) | NA | NA |
| **Charlson Comorbidity Index (categories)** | | | | |
| 0 | 219 (2.7) | 844 (2.6) | 1.00 | reference |
| 1-3 | 7,794 (95.7) | 31,216 (96.3) | 0.97 | 0.85-1.11 |
| 4-6 | 131 (1.6) | 367 (1.1) | 1.39 | 1.10-1.74 |
| 7-8 | X | X | NA | NA |

Characteristics and co-medication of cases with **peptic or esophageal ulcer** and matched controls at the index date

| | No. of Cases (%) (N=835) | No. of Controls (%) (N=3,328) | Crude OR | 95% CI |
|---|---|---|---|---|
| Male | 332 (39.8) | 1,316 (39.5) | NA | NA |
| Female | 503 (60.2) | 2,012 (60.5) | NA | NA |
| **Age** | | | | |
| 18-39 | 87 (10.4) | 343 (10.3) | NA | NA |
| 40-59 | 239 (28.6) | 966 (29.0) | NA | NA |
| 60-79 | 359 (43.0) | 1,435 (43.1) | NA | NA |
| ≥80 | 150 (18.0) | 584 (17.6) | NA | NA |
| **Smoking status** | | | | |
| non | 363 (43.5) | 1,616 (48.6) | 1.00 | reference |
| current | 141 (16.9) | 379 (11.4) | 1.70 | 1.35-2.13 |
| ex | 316 (37.8) | 1,264 (38.0) | 1.11 | 0.93-1.32 |
| unknown | 15 (1.8) | 69 (2.1) | 0.95 | 0.53-1.70 |
| **Alcohol consumption (average units per week)** | | | | |
| none | 440 (52.7) | 1,537 (46.2) | 1.00 | reference |
| <14 | 246 (29.5) | 1,163 (35.0) | 0.73 | 0.62-0.87 |
| ≥14 | 97 (11.6) | 350 (10.5) | 0.96 | 0.74-1.25 |
| unknown | 52 (6.2) | 278 (8.4) | 0.64 | 0.47-0.88 |
| **Body mass index, kg/m$^2$** | | | | |
| <18.5 | 17 (2.0) | 37 (1.1) | 1.98 | 1.09-3.58 |
| 18.5-24.9 | 242 (29.0) | 1,032 (31.0) | 1.00 | reference |
| 25-29.9 | 294 (35.2) | 1,120 (33.7) | 1.13 | 0.93-1.37 |
| ≥30 | 230 (27.5) | 863 (25.9) | 1.16 | 0.94-1.42 |
| unknown | 52 (6.2) | 276 (8.3) | 0.79 | 0.57-1.10 |
| **Concomitant[a] drug use** | | | | |
| SABA | 691 (82.8) | 2,755 (82.8) | 0.94 | 0.59-1.49 |
| SAMA | 50 (6.0) | 185 (5.6) | 1.10 | 0.79-1.53 |
| ICS (mono preparations) | 604 (72.3) | 2,522 (75.8) | 1.00 | 0.67-1.51 |
| ICS+LABA | 283 (33.9) | 993 (29.8) | 1.26 | 1.06-1.51 |
| Asthma add-on therapy[b] | 47 (5.6) | 106 (3.2) | 1.83 | 1.29-2.61 |
| Platelet aggregation inhibitors | 245 (29.3) | 641 (19.3) | 2.05 | 1.69-2.50 |
| Anticoagulants | 57 (6.8) | 123 (3.7) | 1.96 | 1.41-2.73 |
| Proton pump inhibitors | 388 (46.5) | 708 (21.3) | 3.93 | 3.29-4.70 |
| Immunosuppressants[c] | X | 7 (0.2) | NA | NA |
| Bisphosphonates | 51 (6.1) | 125 (3.8) | 1.74 | 1.23-2.46 |
| Vitamin D | 71 (8.5) | 175 (5.3) | 1.78 | 1.32-2.41 |
| **Charlson Comorbidity Index** | | | | |
| Median (25% perc./75% perc./range) | 1 (1/2/12) | 1 (1/1/9) | NA | NA |
| **Charlson Comorbidity Index (categories)** | | | | |
| 0 | 16 (1.9) | 77 (2.3) | 1.00 | reference |
| 1-3 | 743 (89.0) | 3,072 (92.3) | 1.20 | 0.71-2.03 |
| 4-6 | 71 (8.5) | 169 (5.1) | 2.14 | 1.19-3.84 |
| 7-12 | 5 (0.6) | 10 (0.3) | 2.54 | 0.93-6.99 |
| **Number of prescriptions ICS prior** | | | | |
| Median (25% perc./75% perc./range) | 35 (15/66/312) | 33 (14/64/281) | NA | NA |

Characteristics and co-medication of cases with **severe infections** and matched controls at the index date

| | No. of Cases (%) (N=16,160) | No. of Controls (%) (N=64,316) | Crude OR | 95% CI |
|---|---|---|---|---|
| Male | 5,223 (32.3) | 20,709 (32.2) | NA | NA |
| Female | 10,937 (67.7) | 43,607 (67.8) | NA | NA |
| **Age** | | | | |
| 18-39 | 4,976 (30.8) | 19,901 (30.9) | NA | NA |
| 40-59 | 5,084 (31.5) | 20,337 (31.6) | NA | NA |
| 60-79 | 4,629 (28.6) | 18,499 (28.8) | NA | NA |
| ≥80 | 1,471 (9.1) | 5,579 (8.7) | NA | NA |
| **Smoking status** | | | | |
| non | 7,457 (46.1) | 33,128 (51.1) | 1.00 | reference |
| current | 2,981 (18.5) | 9,512 (14.8) | 1.41 | 1.34-1.48 |
| ex | 5,479 (33.9) | 20,441 (31.8) | 1.19 | 1.14-1.24 |
| unknown | 243 (1.5) | 1,235 (1.9) | 0.86 | 0.74-0.99 |
| **Alcohol consumption (average units per week)** | | | | |
| none | 8,050 (49.8) | 29,212 (45.4) | 1.00 | reference |
| <14 | 5,012 (31.0) | 21,137 (32.9) | 0.86 | 0.82-0.89 |
| ≥14 | 1,450 (9.0) | 6,400 (10.0) | 0.81 | 0.76-0.86 |
| unknown | 1,648 (10.2) | 7,567 (11.8) | 0.77 | 0.73-0.82 |
| **Body mass index, kg/m$^2$** | | | | |
| <18.5 | 345 (2.1) | 1,115 (1.7) | 1.33 | 1.18-1.51 |
| 18.5-24.9 | 4,730 (29.3) | 20,861 (32.4) | 1.00 | reference |
| 25-29.9 | 4,883 (30.2) | 19,793 (30.8) | 1.10 | 1.05-1.15 |
| ≥30 | 4,964 (30.7) | 16,182 (25.2) | 1.38 | 1.32-1.45 |
| unknown | 1,238 (7.7) | 6,365 (9.9) | 0.82 | 0.76-0.88 |
| **Concomitant[a] drug use** | | | | |
| SABA | 14,133 (87.5) | 54,432 (84.6) | 1.37 | 1.19-1.58 |
| SAMA | 694 (4.3) | 1,598 (2.5) | 1.84 | 1.68-2.02 |
| ICS (mono preparations) | 11,211 (69.4) | 46,924 (73.0) | 0.90 | 0.83-0.97 |
| ICS+LABA | 6,145 (38.0) | 20,649 (32.1) | 1.38 | 1.33-1.44 |
| Asthma add-on therapy[b] | 942 (5.8) | 2,509 (3.9) | 1.56 | 1.45-1.69 |
| Platelet aggregation inhibitors | 2,570 (15.9) | 6,938 (10.8) | 1.84 | 1.74-1.94 |
| Anticoagulants | 632 (3.9) | 1,299 (2.0) | 2.04 | 1.85-2.26 |
| Proton pump inhibitors | 4,458 (27.6) | 11,064 (17.2) | 2.13 | 2.03-2.22 |
| Immunosuppressants[c] | 61 (0.4) | 84 (0.1) | 2.95 | 2.12-4.11 |
| Bisphosphonates | 592 (3.7) | 1,545 (2.4) | 1.62 | 1.46-1.79 |
| Vitamin D | 974 (6.0) | 2,469 (3.8) | 1.71 | 1.58-1.86 |
| **Charlson Comorbidity Index** | | | | |
| Median (25% perc./75% perc./range) | 1 (1/1/9) | 1 (1/1/9) | NA | NA |
| **Charlson Comorbidity Index (categories)** | | | | |
| 0 | 334 (2.1) | 1,469 (2.3) | 1.00 | reference |
| 1-3 | 14,927 (92.4) | 60,848 (94.6) | 1.09 | 0.98-1.22 |
| 4-6 | 839 (5.2) | 1,921 (3.0) | 2.01 | 1.77-2.30 |
| 7-9 | 60 (0.4) | 78 (0.1) | 3.62 | 2.65-4.94 |
| **Number of prescriptions ICS prior** | | | | |
| Median (25% perc./75% perc./range) | 26 (10/54/335) | 25 (10/51/528) | NA | NA |

Characteristics and co-medication of cases with **herpes zoster** and matched controls at the index date

| | No. of Cases (%) (N=4,163) | No. of Controls (%) (N=16,605) | Crude OR | 95% CI |
|---|---|---|---|---|
| Male | 1,414 (34.0) | 5,621 (33.9) | NA | NA |
| Female | 2,749 (66.0) | 10,984 (66.2) | NA | NA |
| **Age** | | | | |
| 18-39 | 729 (17.5) | 2,923 (17.6) | NA | NA |
| 40-59 | 1,353 (32.5) | 5,402 (32.5) | NA | NA |
| 60-79 | 1,717 (41.2) | 6,884 (41.5) | NA | NA |
| ≥80 | 364 (8.7) | 1,396 (8.4) | NA | NA |
| **Smoking status** | | | | |
| non | 2,118 (50.9) | 8,403 (50.6) | 1.00 | reference |
| current | 525 (12.6) | 2,234 (13.5) | 0.93 | 0.83-1.03 |
| ex | 1,466 (35.2) | 5,624 (33.9) | 1.04 | 0.96-1.12 |
| unknown | 54 (1.3) | 344 (2.1) | 0.60 | 0.44-0.81 |
| **Alcohol consumption (average units per week)** | | | | |
| none | 1,931 (46.4) | 7,996 (48.2) | 1.00 | reference |
| <14 | 1,410 (33.9) | 5,388 (32.5) | 1.09 | 1.01-1.17 |
| ≥14 | 445 (10.7) | 1,654 (10.0) | 1.13 | 1.00-1.27 |
| unknown | 377 (9.1) | 1,567 (9.4) | 1.00 | 0.88-1.14 |
| **Body mass index, kg/m$^2$** | | | | |
| <18.5 | 63 (1.5) | 203 (1.2) | 1.21 | 0.90-1.61 |
| 18.5-24.9 | 1,304 (31.3) | 5,060 (30.5) | 1.00 | reference |
| 25-29.9 | 1,339 (32.2) | 5,377 (32.4) | 0.97 | 0.88-1.05 |
| ≥30 | 1,130 (27.1) | 4,528 (27.3) | 0.97 | 0.88-1.06 |
| unknown | 327 (7.9) | 1,437 (8.7) | 0.87 | 0.76-1.01 |
| **Concomitant[a] drug use** | | | | |
| SABA | 3,452 (82.9) | 13,901 (83.7) | 0.88 | 0.70-1.11 |
| SAMA | 156 (3.8) | 620 (3.7) | 1.01 | 0.84-1.22 |
| ICS (mono preparations) | 3,004 (72.2) | 12,224 (73.6) | 0.99 | 0.85-1.17 |
| ICS+LABA | 1,416 (34.0) | 5,250 (31.6) | 1.15 | 1.07-1.25 |
| Asthma add-on therapy[b] | 215 (5.2) | 703 (4.2) | 1.24 | 1.06-1.46 |
| Platelet aggregation inhibitors | 650 (15.6) | 2,337 (14.1) | 1.16 | 1.05-1.29 |
| Anticoagulants | 149 (3.6) | 491 (3.0) | 1.22 | 1.01-1.48 |
| Proton pump inhibitors | 1,050 (25.2) | 3,517 (21.2) | 1.32 | 1.21-1.44 |
| Immunosuppressants[c] | 17 (0.4) | 37 (0.2) | 1.84 | 1.04-3.26 |
| Bisphosphonates | 151 (3.6) | 542 (3.3) | 1.13 | 0.93-1.36 |
| Vitamin D | 210 (5.0) | 840 (5.1) | 0.99 | 0.85-1.17 |
| **Charlson Comorbidity Index** | | | | |
| Median (25% perc./75% perc./range) | 1 (1/1/10) | 1 (1/1/8) | NA | NA |
| **Charlson Comorbidity Index (categories)** | | | | |
| 0 | 88 (2.1) | 385 (2.3) | 1.00 | reference |
| 1-3 | 3,877 (93.1) | 15,595 (93.9) | 1.10 | 0.88-1.36 |
| 4-6 | 187 (4.5) | 595 (3.6) | 1.38 | 1.06-1.79 |
| 7-10 | 11 (0.3) | 30 (0.2) | 1.60 | 0.85-3.04 |
| **Number of prescriptions ICS prior** | | | | |
| Median (25% perc./75% perc./range) | 29 (12/57/269) | 29 (12/56/528) | NA | NA |

Characteristics and co-medication of cases with **type 2 diabetes mellitus** and matched controls at the index date

| | No. of Cases (%) (N=5,189) | No. of Controls (%) (N=20,715) | Crude OR | 95% CI |
|---|---|---|---|---|
| Male | 2,070 (39.9) | 8,256 (39.9) | NA | NA |
| Female | 3,119 (60.1) | 12,459 (60.1) | NA | NA |
| **Age** | | | | |
| 18-39 | 653 (12.6) | 2,609 (12.6) | NA | NA |
| 40-59 | 1,927 (37.1) | 7,728 (37.3) | NA | NA |
| 60-79 | 2,252 (43.4) | 8,984 (43.4) | NA | NA |
| ≥80 | 357 (6.9) | 1,394 (6.7) | NA | NA |
| **Smoking status** | | | | |
| non | 2,313 (44.6) | 10,373 (50.1) | 1.00 | reference |
| current | 751 (14.5) | 2,619 (12.6) | 1.29 | 1.18-1.42 |
| ex | 2,054 (39.6) | 7,318 (35.3) | 1.27 | 1.19-1.36 |
| unknown | 71 (1.4) | 405 (2.0) | 0.76 | 0.58-0.99 |
| **Alcohol consumption (average units per week)** | | | | |
| none | 2,936 (56.6) | 9,296 (44.9) | 1.00 | reference |
| <14 | 1,497 (28.9) | 7,079 (34.2) | 0.66 | 0.62-0.71 |
| ≥14 | 519 (10.0) | 2,490 (12.0) | 0.63 | 0.57-0.71 |
| unknown | 237 (4.6) | 1,850 (8.9) | 0.39 | 0.33-0.45 |
| **Body mass index, kg/m$^2$** | | | | |
| <18.5 | 15 (0.3) | 266 (1.3) | 0.72 | 0.42-1.23 |
| 18.5-24.9 | 464 (8.9) | 6,267 (30.3) | 1.00 | reference |
| 25-29.9 | 1,294 (24.9) | 7,241 (35.0) | 2.47 | 2.21-2.77 |
| ≥30 | 3,180 (61.3) | 5,312 (25.6) | 8.88 | 7.96-9.89 |
| unknown | 236 (4.6) | 1,629 (7.9) | 1.81 | 1.52-2.15 |
| **Concomitant[a] drug use** | | | | |
| SABA | 4,485 (86.4) | 17,037 (82.2) | 1.32 | 1.05-1.65 |
| SAMA | 239 (4.6) | 676 (3.3) | 1.49 | 1.27-1.74 |
| ICS (mono preparations) | 3,647 (70.3) | 15,030 (72.6) | 1.02 | 0.88-1.18 |
| ICS+LABA | 1,879 (36.2) | 6,805 (32.9) | 1.19 | 1.11-1.28 |
| Asthma add-on therapy[b] | 259 (5.0) | 931 (4.5) | 1.13 | 0.98-1.30 |
| Platelet aggregation inhibitors | 998 (19.2) | 2,541 (12.3) | 1.92 | 1.76-2.10 |
| Anticoagulants | 249 (4.8) | 569 (2.8) | 1.84 | 1.58-2.15 |
| Proton pump inhibitors | 1,514 (29.2) | 4,218 (20.4) | 1.74 | 1.61-1.87 |
| Immunosuppressants[c] | 21 (0.4) | 44 (0.2) | 1.91 | 1.13-3.20 |
| Bisphosphonates | 119 (2.3) | 639 (3.1) | 0.72 | 0.58-0.88 |
| Vitamin D | 238 (4.6) | 929 (4.5) | 1.03 | 0.89-1.20 |
| **Charlson Comorbidity Index** | | | | |
| Median (25% perc./75% perc./range) | 1 (1/1/6) | 1(1/1/7) | NA | NA |
| **Charlson Comorbidity Index (categories)** | | | | |
| 0 | 122 (2.4) | 511 (2.5) | 1.00 | reference |
| 1-3 | 4,963 (95.6) | 19,932 (96.2) | 1.05 | 0.87-1.26 |
| 4-6 | 104 (2.0) | 268 (1.3) | 1.65 | 1.25-2.17 |
| 7 | X | X | NA | NA |
| **Number of prescriptions ICS prior** | | | | |
| Median (25% perc./75% perc./range) | 31 (13/59/372) | 29 (12/55/604) | NA | NA |

Characteristics and co-medication of cases with **cataract** and matched controls at the index date

| | No. of Cases (%) (N=5,327) | No. of Controls (%) (N=21,019) | Crude OR | 95% CI |
|---|---|---|---|---|
| Male | 1,659 (31.1) | 6,507 (31.0) | NA | NA |
| Female | 3,668 (68.9) | 14,512 (69.0) | NA | NA |
| **Age** | | | | |
| 18-39 | 50 (0.9) | 195 (0.9) | NA | NA |
| 40-59 | 536 (10.1) | 2,157 (10.3) | NA | NA |
| 60-79 | 3,240 (60.8) | 12,975 (61.7) | NA | NA |
| ≥80 | 1,501 (28.2) | 5,692 (27.1) | NA | NA |
| **Smoking status** | | | | |
| non | 2,524 (47.4) | 10,739 (51.1) | 1.00 | reference |
| current | 382 (7.2) | 1,454 (6.9) | 1.12 | 0.99-1.27 |
| ex | 2,333 (43.8) | 8,388 (39.9) | 1.19 | 1.12-1.27 |
| unknown | 88 (1.7) | 438 (2.1) | 0.84 | 0.66-1.07 |
| **Alcohol consumption (average units per week)** | | | | |
| none | 2,892 (54.3) | 11,123 (52.9) | 1.00 | reference |
| <14 | 1,623 (30.5) | 6,480 (30.8) | 0.97 | 0.90-1.03 |
| ≥14 | 475 (8.9) | 1,865 (8.9) | 0.98 | 0.87-1.10 |
| unknown | 337 (6.3) | 1,551 (7.4) | 0.83 | 0.73-0.94 |
| **Body mass index, kg/m$^2$** | | | | |
| <18.5 | 90 (1.7) | 266 (1.3) | 1.34 | 1.05-1.72 |
| 18.5-24.9 | 1,588 (29.8) | 6,316 (30.1) | 1.00 | reference |
| 25-29.9 | 1,898 (35.6) | 7,572 (36.0) | 1.00 | 0.93-1.08 |
| ≥30 | 1,425 (26.8) | 5,394 (25.7) | 1.06 | 0.98-1.15 |
| unknown | 326 (6.1) | 1,471 (7.0) | 0.88 | 0.76-1.00 |
| **Concomitant[a] drug use** | | | | |
| SABA | 4,394 (82.5) | 16,904 (80.4) | 1.27 | 1.05-1.55 |
| SAMA | 338 (6.4) | 1,274 (6.1) | 1.07 | 0.94-1.21 |
| ICS (mono preparations) | 3,756 (70.5) | 15,278 (72.7) | 0.90 | 0.78-1.04 |
| ICS+LABA | 1,877 (35.2) | 6,878 (32.7) | 1.15 | 1.07-1.23 |
| Asthma add-on therapy[b] | 233 (4.4) | 765 (3.6) | 1.23 | 1.06-1.43 |
| Platelet aggregation inhibitors | 1,623 (30.5) | 5,193 (24.7) | 1.39 | 1.30-1.50 |
| Anticoagulants | 343 (6.4) | 1,104 (5.3) | 1.23 | 1.08-1.39 |
| Proton pump inhibitors | 1,720 (32.3) | 5,870 (27.9) | 1.27 | 1.18-1.36 |
| Immunosuppressants[c] | 25 (0.5) | 34 (0.2) | 2.89 | 1.70-4.90 |
| Bisphosphonates | 370 (7.0) | 1,302 (6.2) | 1.14 | 1.01-1.29 |
| Vitamin D | 540 (10.4) | 1,842 (8.8) | 1.18 | 1.06-1.31 |
| **Charlson Comorbidity Index** | | | | |
| Median (25% perc./75% perc./range) | 1 (1/2/10) | 1 (1/2/9) | NA | NA |
| **Charlson Comorbidity Index (categories)** | | | | |
| 0 | 78 (1.5) | 387 (1.8) | 1.00 | reference |
| 1-3 | 4,670 (87.7) | 19,255 (91.6) | 1.23 | 0.98-1.53 |
| 4-6 | 546 (10.3) | 1,327 (6.3) | 2.09 | 1.64-2.65 |
| 7-10 | 33 (0.6) | 50 (0.2) | 3.40 | 2.21-5.21 |
| **Number of prescriptions ICS** | | | | |
| Median (25% perc./75% perc./range) | 41 (21/72/296) | 40 (20/69/350) | NA | NA |

Characteristics and co-medication of cases with **glaucoma** and matched controls at the index date

| | No. of Cases (%) (N=1,467) | No. of Controls (%) (N=5,832) | Crude OR | 95% CI |
|---|---|---|---|---|
| Male | 544 (37.1) | 2,156 (37.0) | NA | NA |
| Female | 923 (62.9) | 3,676 (63.0) | NA | NA |
| **Age** | | | | |
| 18-39 | 42 (2.9) | 171 (2.9) | NA | NA |
| 40-59 | 302 (20.6) | 1,206 (20.7) | NA | NA |
| 60-79 | 832 (56.7) | 3,335 (57.2) | NA | NA |
| ≥80 | 291 (19.8) | 1,120 (19.2) | NA | NA |
| **Smoking status** | | | | |
| non | 753 (51.3) | 2,854 (48.9) | 1.00 | reference |
| current | 132 (9.0) | 543 (9.3) | 0.92 | 0.75-1.14 |
| ex | 552 (37.6) | 2,279 (39.1) | 0.91 | 0.80-1.03 |
| unknown | 30 (2.0) | 156 (2.7) | 0.72 | 0.48-1.09 |
| **Alcohol consumption (average units per week)** | | | | |
| none | 738 (50.3) | 2,913 (50.0) | 1.00 | reference |
| <14 | 481 (32.8) | 1,859 (31.9) | 1.03 | 0.90-1.17 |
| ≥14 | 151 (10.3) | 590 (10.1) | 1.02 | 0.83-1.25 |
| unknown | 97 (6.6) | 470 (8.1) | 0.82 | 0.65-1.03 |
| **Body mass index, kg/m$^2$** | | | | |
| <18.5 | 21 (1.4) | 68 (1.2) | 1.22 | 0.74-2.01 |
| 18.5-24.9 | 447 (30.5) | 1,756 (30.1) | 1.00 | reference |
| 25-29.9 | 513 (35.0) | 2,029 (34.8) | 1.00 | 0.86-1.15 |
| ≥30 | 393 (26.8) | 1,524 (26.1) | 1.02 | 0.88-1.20 |
| unknown | 93 (6.3) | 455 (7.8) | 0.79 | 0.62-1.02 |
| **Concomitant[a] drug use** | | | | |
| SABA | 1,181 (80.5) | 4,748 (81.4) | 0.81 | 0.58-1.11 |
| SAMA | 98 (6.7) | 346 (5.9) | 1.13 | 0.89-1.44 |
| ICS (mono preparations) | 1,083 (73.8) | 4,270 (73.2) | 1.00 | 0.75-1.33 |
| ICS+LABA | 451 (30.7) | 1,876 (32.2) | 0.94 | 0.82-1.08 |
| Asthma add-on therapy[b] | 67 (4.6) | 211 (3.6) | 1.26 | 0.95-1.68 |
| Platelet aggregation inhibitors | 373 (25.4) | 1,268 (21.7) | 1.28 | 1.11-1.47 |
| Anticoagulants | 69 (4.7) | 249 (4.3) | 1.08 | 0.82-1.43 |
| Proton pump inhibitors | 389 (26.5) | 1,487 (25.5) | 1.04 | 0.90-1.19 |
| Immunosuppressants[c] | 6 (0.4) | 18 (0.3) | 1.33 | 0.53-3.36 |
| Bisphosphonates | 61 (4.2) | 294 (5.0) | 0.81 | 0.60-1.07 |
| Vitamin D | 94 (6.4) | 390 (6.7) | 0.96 | 0.76-1.22 |
| **Charlson Comorbidity Index** | | | | |
| Median (25% perc./75% perc./range) | 1 (1/2/8) | 1 (1/2/10) | NA | NA |
| **Charlson Comorbidity Index (categories)** | | | | |
| 0 | 30 (2.0) | 120 (2.1) | 1.00 | reference |
| 1-3 | 1,333 (90.9) | 5,368 (92.0) | 1.00 | 0.69-1.44 |
| 4-6 | 99 (6.8) | 326 (5.6) | 1.20 | 0.79-1.83 |
| 7-10 | 5 (0.3) | 18 (0.3) | 1.13 | 0.44-2.92 |
| **Number of prescriptions ICS prior** | | | | |
| Median (25% perc./75% perc./range) | 33 (15/60/241) | 36 (16/66/351) | NA | NA |

Characteristics and co-medication of cases with **chronic kidney disease** and matched controls at the index date

| | No. of Cases (%) (N=8,778) | No. of Controls (%) (N=34,762) | Crude OR | 95% CI |
|---|---|---|---|---|
| Male | 2,364 (26.9) | 9,254 (26.6) | NA | NA |
| Female | 6,414 (73.1) | 25,508 (73.4) | NA | NA |
| **Age** | | | | |
| 18-39 | 170 (1.9) | 668 (1.9) | NA | NA |
| 40-59 | 1,062 (12.1) | 4,260 (12.3) | NA | NA |
| 60-79 | 5,158 (58.8) | 20,727 (59.6) | NA | NA |
| ≥80 | 2,388 (27.2) | 9,107 (26.2) | NA | NA |
| **Smoking status** | | | | |
| non | 4,132 (47.1) | 17,767 (51.1) | 1.00 | reference |
| current | 622 (7.1) | 2,710 (7.8) | 0.99 | 0.90-1.09 |
| ex | 3,925 (44.7) | 13,713 (39.5) | 1.24 | 1.18-1.31 |
| unknown | 99 (1.1) | 572 (1.7) | 0.73 | 0.58-0.91 |
| **Alcohol consumption (average units per week)** | | | | |
| none | 5,172 (58.9) | 17,924 (51.6) | 1.00 | reference |
| <14 | 2,633 (30.0) | 11,103 (31.9) | 0.82 | 0.77-0.86 |
| ≥14 | 565 (6.4) | 3,024 (8.7) | 0.63 | 0.57-0.70 |
| unknown | 408 (4.7) | 2,711 (7.8) | 0.52 | 0.46-0.58 |
| **Body mass index, kg/m$^2$** | | | | |
| <18.5 | 104 (1.2) | 532 (1.5) | 1.00 | 0.80-1.24 |
| 18.5-24.9 | 2,061 (23.5) | 10,893 (31.3) | 1.00 | reference |
| 25-29.9 | 3,140 (35.8) | 12,285 (35.3) | 1.39 | 1.31-1.48 |
| ≥30 | 3,074 (35.0) | 8,432 (24.3) | 2.03 | 1.90-2.17 |
| unknown | 399 (4.6) | 2,620 (7.5) | 0.77 | 0.69-0.87 |
| **Concomitant[a] drug use** | | | | |
| SABA | 7,241 (82.5) | 28,138 (80.9) | 0.94 | 0.82-1.09 |
| SAMA | 662 (7.5) | 2,015 (5.8) | 1.34 | 1.23-1.48 |
| ICS (mono preparations) | 6,390 (72.8) | 25,978 (74.7) | 0.79 | 0.70-0.88 |
| ICS+LABA | 2,981 (34.0) | 10,886 (31.3) | 1.14 | 1.08-1.20 |
| Asthma add-on therapy[b] | 346 (3.9) | 1,320 (3.8) | 1.04 | 0.92-1.17 |
| Platelet aggregation inhibitors | 3,188 (36.3) | 7,773 (22.4) | 2.21 | 2.09-2.33 |
| Anticoagulants | 710 (8.1) | 1,352 (3.9) | 2.22 | 2.02-2.45 |
| Proton pump inhibitors | 2,952 (33.6) | 8,554 (24.6) | 1.61 | 1.53-1.70 |
| Immunosuppressants[c] | 35 (0.4) | 66 (0.2) | 2.10 | 1.40-3.17 |
| Bisphosphonates | 548 (6.2) | 2,153 (6.2) | 1.00 | 0.90-1.10 |
| Vitamin D | 805 (9.2) | 2,774 (8.0) | 1.18 | 1.08-1.28 |
| **Charlson Comorbidity Index** | | | | |
| Median (25% perc./75% perc./range) | 1 (1/2/8) | 1 (1/1/8) | NA | NA |
| **Charlson Comorbidity Index (categories)** | | | | |
| 0 | 183 (2.1) | 786 (2.3) | 1.00 | reference |
| 1-3 | 8,004 (91.2) | 33,166 (95.4) | 1.04 | 0.90-1.21 |
| 4-6 | 572 (6.5) | 798 (2.3) | 3.14 | 2.63-3.73 |
| 7-9 | 19 (0.2) | 12 (0.0) | 7.43 | 4.07-13.55 |
| **Number of prescriptions ICS prior** | | | | |
| Median (25% perc./75% perc./range) | 37 (17/66/412) | 36 (17/64/363) | NA | NA |

Characteristics and co-medication of cases with **affective disorders** and matched controls at the index date

| | No. of Cases (%) (N=4,962) | No. of Controls (%) (N=19,809) | Crude OR | 95% CI |
|---|---|---|---|---|
| Male | 1,874 (37.8) | 7,479 (37.8) | NA | NA |
| Female | 3,088 (62.2) | 12,330 (62.2) | NA | NA |
| **Age** | | | | |
| 18-39 | 2,586 (52.1) | 10,316 (52.1) | NA | NA |
| 40-59 | 1,665 (33.6) | 6,657 (33.6) | NA | NA |
| 60-79 | 579 (11.7) | 2,328 (11.8) | NA | NA |
| ≥80 | 132 (2.7) | 508 (2.6) | NA | NA |
| **Smoking status** | | | | |
| non | 2,268 (45.7) | 11,305 (57.1) | 1.00 | reference |
| current | 1,247 (25.1) | 3,129 (15.8) | 2.01 | 1.86-2.18 |
| ex | 1,316 (26.5) | 4,727 (23.9) | 1.38 | 1.28-1.50 |
| unknown | 131 (2.6) | 648 (3.3) | 1.01 | 0.83-1.24 |
| **Alcohol consumption (average units per week)** | | | | |
| none | 2,099 (42.3) | 7,768 (39.2) | 1.00 | reference |
| <14 | 1,613 (32.5) | 6,519 (32.9) | 0.92 | 0.85-0.99 |
| ≥14 | 565 (11.4) | 2,145 (10.8) | 0.97 | 0.87-1.08 |
| unknown | 685 (13.8) | 3,377 (17.1) | 0.73 | 0.66-0.80 |
| **Body mass index, kg/m$^2$** | | | | |
| <18.5 | 127 (2.6) | 387 (2.0) | 1.43 | 1.16-1.76 |
| 18.5-24.9 | 1,627 (32.8) | 7,197 (36.3) | 1.00 | reference |
| 25-29.9 | 1,366 (27.5) | 5,414 (27.3) | 1.13 | 1.04-1.22 |
| ≥30 | 1,202 (24.2) | 3,919 (19.8) | 1.38 | 1.26-1.50 |
| unknown | 640 (12.9) | 2,892 (14.6) | 0.96 | 0.86-1.06 |
| **Concomitant[a] drug use** | | | | |
| SABA | 4,403 (88.7) | 17,039 (86.0) | 1.81 | 1.35-2.44 |
| SAMA | 124 (2.5) | 327 (1.7) | 1.57 | 1.26-1.94 |
| ICS (mono preparations) | 3,692 (74.4) | 15,096 (76.2) | 0.94 | 0.81-1.10 |
| ICS+LABA | 1,602 (32.3) | 5,709 (28.8) | 1.23 | 1.14-1.32 |
| Asthma add-on therapy[b] | 236 (4.8) | 718 (3.6) | 1.34 | 1.15-1.56 |
| Platelet aggregation inhibitors | 304 (6.1) | 805 (4.1) | 1.73 | 1.48-2.01 |
| Anticoagulants | 64 (1.3) | 166 (0.8) | 1.58 | 1.17-2.12 |
| Proton pump inhibitors | 694 (14.0) | 1,685 (8.5) | 1.93 | 1.75-2.14 |
| Immunosuppressants[c] | 8 (0.2) | 27 (0.1) | 1.19 | 0.54-2.61 |
| Bisphosphonates | 64 (1.3) | 180 (0.9) | 1.45 | 1.07-1.95 |
| Vitamin D | 93 (1.9) | 313 (1.6) | 1.21 | 0.95-1.55 |
| **Charlson Comorbidity Index** | | | | |
| Median (25% perc./75% perc./range) | 1 (1/1/10) | 1 (1/1/8) | NA | NA |
| **Charlson Comorbidity Index (categories)** | | | | |
| 0 | 107 (2.2) | 400 (2.0) | 1.00 | reference |
| 1-3 | 4,772 (96.2) | 19,214 (97.0) | 0.93 | 0.77-1.13 |
| 4-6 | 80 (1.6) | 191 (1.0) | 1.60 | 1.18-2.17 |
| 7-10 | X | X | NA | NA |
| **Number of prescriptions ICS prior** | | | | |
| Median (25% perc./75% perc./range) | 21 (8/45/358) | 21 (8/44/348) | NA | NA |

Characteristics and co-medication of cases with **cardiovascular disease** and matched controls at the index date

| | No. of Cases (%) (N=4,014) | No. of Controls (%) (N=15,945) | Crude OR | 95% CI |
|---|---|---|---|---|
| Male | 1,885 (47.0) | 7,477 (46.9) | NA | NA |
| Female | 2,129 (53.0) | 8,468 (53.1) | NA | NA |
| **Age** | | | | |
| 18-39 | 66 (1.6) | 265 (1.7) | NA | NA |
| 40-59 | 977 (24.3) | 3,913 (24.5) | NA | NA |
| 60-79 | 2,274 (56.7) | 9,087 (57.0) | NA | NA |
| ≥80 | 697 (17.4) | 2,680 (16.8) | NA | NA |
| **Smoking status** | | | | |
| non | 1,746 (43.5) | 7,954 (49.9) | 1.00 | reference |
| current | 544 (13.6) | 1,495 (9.4) | 1.70 | 1.51-1.90 |
| ex | 1,622 (40.4) | 6,046 (37.9) | 1.23 | 1.14-1.33 |
| unknown | 102 (2.5) | 450 (2.8) | 1.03 | 0.82-1.29 |
| **Alcohol consumption (average units per week)** | | | | |
| none | 2,048 (51.0) | 7,294 (45.7) | 1.00 | reference |
| <14 | 1,311 (32.7) | 5,243 (32.9) | 0.89 | 0.82-0.96 |
| ≥14 | 427 (10.6) | 1,973 (12.4) | 0.76 | 0.67-0.85 |
| unknown | 228 (5.7) | 1,435 (9.0) | 0.56 | 0.48-0.65 |
| **Body mass index, kg/m$^2$** | | | | |
| <18.5 | 39 (1.0) | 192 (1.2) | 0.96 | 0.68-1.37 |
| 18.5-24.9 | 994 (24.8) | 4,744 (29.8) | 1.00 | reference |
| 25-29.9 | 1,525 (38.0) | 5,762 (36.1) | 1.27 | 1.16-1.39 |
| ≥30 | 1,142 (28.5) | 3,777 (23.7) | 1.47 | 1.33-1.62 |
| unknown | 314 (7.8) | 1,470 (9.2) | 1.00 | 0.87-1.16 |
| **Concomitant[a] drug use** | | | | |
| SABA | 3,368 (83.9) | 12,932 (81.1) | 1.05 | 0.85-1.29 |
| SAMA | 277 (6.9) | 798 (5.0) | 1.44 | 1.25-1.66 |
| ICS (mono preparations) | 3,053 (76.1) | 12,403 (77.8) | 0.89 | 0.75-1.07 |
| ICS+LABA | 1,213 (30.2) | 4,337 (27.2) | 1.22 | 1.12-1.32 |
| Asthma add-on therapy[b] | 160 (4.0) | 542 (3.4) | 1.19 | 0.99-1.43 |
| Platelet aggregation inhibitors | 1,394 (34.7) | 2,091 (13.1) | 4.00 | 3.67-4.36 |
| Anticoagulants | 200 (5.0) | 462 (2.9) | 1.79 | 1.51-2.13 |
| Proton pump inhibitors | 1,226 (30.5) | 3,362 (21.1) | 1.80 | 1.66-1.96 |
| Immunosuppressants[c] | 12 (0.3) | 21 (0.1) | 2.29 | 1.13-4.65 |
| Bisphosphonates | 167 (4.2) | 627 (3.9) | 1.08 | 0.90-1.29 |
| Vitamin D | 269 (6.7) | 872 (5.5) | 1.27 | 1.09-1.47 |
| **Charlson Comorbidity Index** | | | | |
| Median (25% perc./75% perc./range) | 1 (1/2/7) | 1 (1/1/8) | NA | NA |
| **Charlson Comorbidity Index (categories)** | | | | |
| 0 | 101 (2.5) | 398 (2.5) | 1.00 | reference |
| 1-3 | 3,651 (91.0) | 14,974 (93.9) | 0.97 | 0.80-1.19 |
| 4-6 | 249 (6.2) | 562 (3.5) | 1.77 | 1.39-2.24 |
| 7-8 | 13 (0.3) | 11 (0.1) | 4.62 | 2.21-9.68 |
| **Number of prescriptions ICS prior** | | | | |
| Median (25% perc./75% perc./range) | 34 (14/63/257) | 33 (14/60/408) | NA | NA |

**Abbreviations for all characteristics and co-medication tables:** LABA, long-acting beta-adrenoceptor agonists; ICS, inhaled corticosteroids; SABA, short-acting beta-adrenoceptor agonists; SAMA, short-acting muscarinic antagonists; LAMA, long-acting muscarinic antagonists; NA, not applicable; X: cell contained <5 observations (due to confidentiality reasons, we are not allowed to display cells containing <5 observations); perc., percentile.

[a] Last prescription within 365 days before the index date; reference group: non-users of these drug groups.

[b] Theophylline, leucotriene receptor antagonists, or omalizumab.

[c] Selective immunosuppressants, tumor necrosis factor alpha inhibitors, interleukin inhibitors, calcineurin inhibitors, azathioprine, thalidomide, lenalidomide, or methotrexate.
